# Bonding of Hydrogel to Biometal Surfaces: Principles, Methods and Applications

**DOI:** 10.1002/EXP.20240049

**Published:** 2025-05-07

**Authors:** Yujie Zhou, Luyao Zhang, Shufei Liu, Yan Wei, Yinchun Hu, Xiaojie Lian, Longfei Wang, Ziwei Liang, Weiyi Chen, Xin Xie, Di Huang

**Affiliations:** ^1^ Department of Biomedical Engineering Research Center for Nano‐Biomaterials and Regenerative Medicine College of Biomedical Engineering Taiyuan University of Technology Taiyuan P. R. China; ^2^ Shanxi‐Zheda Institute of Advanced Materials and Chemical Engineering Taiyuan P. R. China; ^3^ Xellar Biosystems Cambridge Massachusetts USA

**Keywords:** biometal, coating method, hydrogel coating application, hydrogel coating

## Abstract

Metal surface coating modification is an effective method to solve the problem of corrosion and inflammation in biometal clinical applications. Hydrogel is currently a commonly used biometal surface coating material. Because of its hydrophilicity, biocompatibility, and good biomechanical properties, hydrogel is widely used in clinical applications. Functionalized hydrogel coatings on biometal surfaces can effectively ameliorate problems such as corrosion, late thrombosis, inflammation, and other complications of implanted metals. Therefore, realizing a strong bond between biometal and hydrogel is a hot issue. This article centers on the bonding of hydrogel to biometal, focusing on a review of (i) biometal surface pretreatment methods, (ii) biometal‐hydrogel bonding methods, and (iii) application of hydrogel coatings on biometal surfaces.

## Graphical abstract

This article reviews the research progress of hydrogel coatings on biometal surfaces, focusing on biometal surface pretreatment methods, hydrogel‐biometal bonding methods, and applications of hydrogel coatings on biometal surfaces. The article also discusses potential future applications of hydrogel coatings on biometal surfaces to provide a broader perspective on the trajectory of the field.

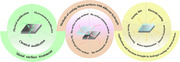

## 1 Introduction

Biometal materials are metals or alloys used in biomedical materials, belonging to a class of inert materials with high fatigue resistance and mechanical strength. These biometals are widely used as load‐bearing implant materials in clinical practice. In the clinical, titanium‐based alloys [1, 2], magnesium‐based alloys [3, 4], cobalt‐based alloys [5, 6], stainless steel [7], shape memory alloys [8], precious metals [9, 10, 11, 12, 13], and so on are commonly used biometal materials. However, the metals and alloys currently used in clinical practice suffer from corrosion‐induced metallosis [14], late thrombosis, inflammation [15], potential sources of infection, and other complications [16]. Therefore, how to modify the biometal surface has become a great concern during the past decades [17, 18].

**FIGURE 1:**
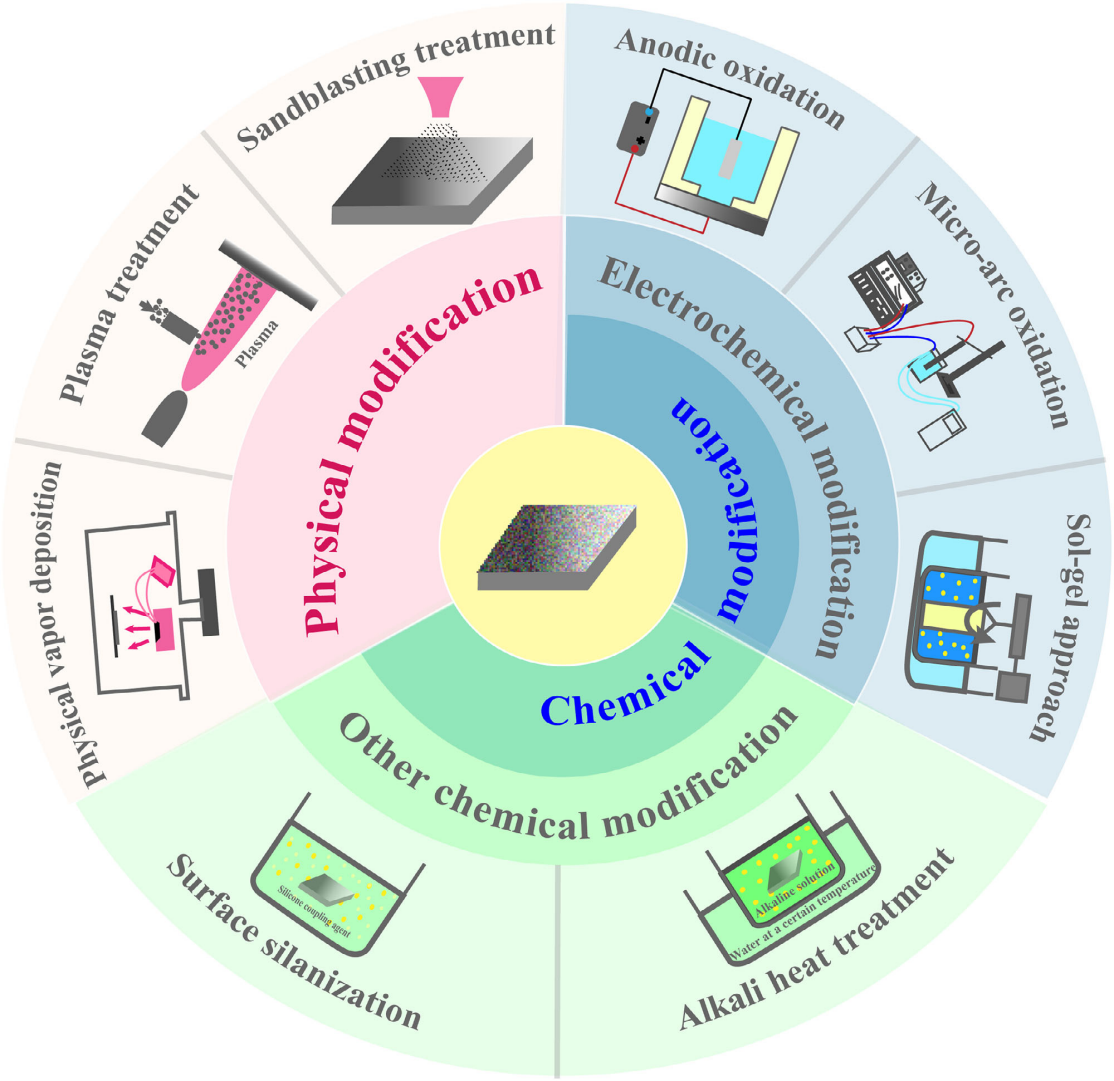
Methods for the surface treatment of biometals and schematic diagrams of various methods. Biometal surface modification methods mainly include physical modification (sandblasting, plasma treatment, physical vapor deposition technology, etc.), chemical modification (electrochemical modification (anodic oxidation, micro‐arc oxidation, sol–gel method, etc.), surface silanization, alkali heat treatment, etc.).

**FIGURE 2:**
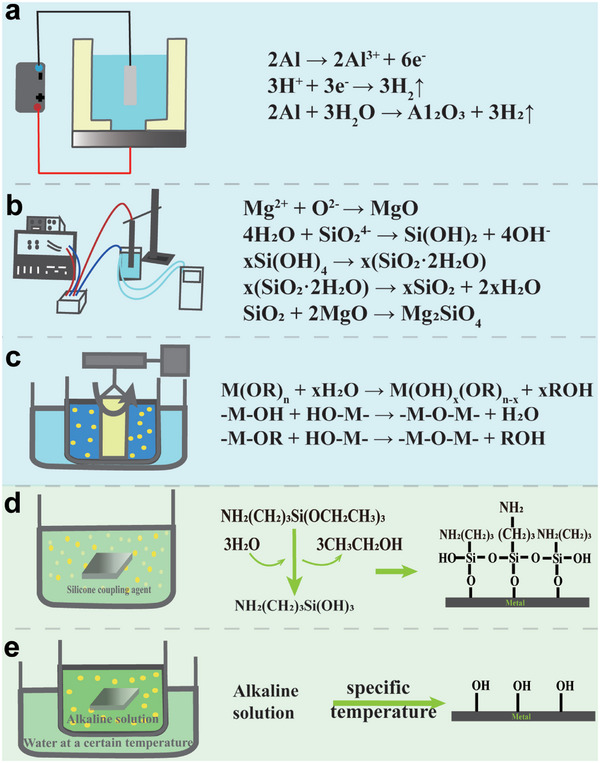
Schematic representation of the chemical reactions and results involved in chemical modification. (a) Anodic oxidation (aluminum as an example). (b) Micro‐arc oxidation (magnesium as an example). Reproduced with permission [27]. Copyright 2022, Elsevier. (c) Sol–gel method. (d) Surface silanization and modification result (3‐aminopropyltriethoxysilane as an example). (e) Alkali heat treatment modification result.

Biometal surface modification methods (Figure 1) mainly include physical modification (sandblasting [19], plasma treatment [20], physical vapor deposition technology [21], etc.), chemical modification (electrochemical modification [anodic oxidation] [22] [Figure 2a], micro‐arc oxidation [23] [Figure 2b], sol–gel method [24] (Figure 2c), etc.), surface silanization [25] [Figure 2d], alkali heat treatment [26] [Figure 2e], and so on). Surface modification is the first step in the complex surface treatment of biometal to give them a different appearance. Different treatment methods have their advantages and disadvantages (Table 1), and in practice, the use of a single surface modification method often fails to achieve multifunctionality such as anti‐inflammation and anti‐corrosion. Therefore, there is a need for a combination of modification methods that can change the surface properties of biometal and, at the same time, confer multifunctionality to biometal.

**TABLE 1:** Analysis of advantages, disadvantages, and applications of biometal surface modification methods.

Method		Metal	Advantages and disadvantages	Applications	References
Physical modification	Sandblasting treatment	Al, Zn, steel, stainless steel, alloys, etc.	To obtain a certain degree of cleanliness and different roughness on the surface of the metal. High noise and pollution.	Suitable for pre‐treatment of metals, etc., in large specialized factories.	[28, 29]
	Plasma treatment	Ti, Ti6Al4V, Ge, etc.	The high energy of the reaction process and the high efficiency of modification. Reaction time is not easy to control.	It is widely used for metal treatment in aerospace, petrochemical, automotive, and other industries.	[20, 30, 31, 32, 33]
	Physical vapor deposition	Al, Ag, Ti6Al4V, etc.	Lower processing temperature and faster deposition rate. High cost of equipment and high technical requirements for operation and maintenance.	Suitable for the preparation of coatings with specific properties (electrical conductivity, abrasion resistance, etc.).	[34, 35, 36]
Electrochemical modification	Anodic oxidation	Ti, TiO_2_, Al, Mg, etc.	Improves various properties of metals, including wear resistance, corrosion resistance. Thickness is not easily controlled.	Used to enhance the corrosion resistance and extend the service life of metals.	[37, 38, 39]
	Micro‐arc oxidation	Ti, TiO_2_, Al, Mg, etc.	The surface hardness of the material is greatly improved, the process is stable and reliable, and the equipment is simple. High energy consumption.	For the formation of hard and thick porous oxide layers on metal substrates.	[40, 41, 42, 43, 44]
	Sol–gel approach	Cu, Ti6Al4V, TC4, etc.	Uniform surface modification. Harmful ingredients, long time required.	Used to form multifunctional coatings on metal surfaces.	[45, 46]
Chemical modification	Surface silanization	Fe, Zn, Al, etc.	Low energy consumption, no heavy metals. Unstable.	Used for grafting stable chemical bonds on metal surfaces.	[47, 48, 49]
	Alkali heat treatment	Ti, Fe, etc.	Enhanced surface hydrophilicity and biocompatibility. High energy consumption.	Used to form biologically active surfaces on metal substrates.	[50, 51, 52]

Hydrogel is now a multi‐functional material for biometal surface coatings. Hydrogels perform endogenous 3D crosslinked networks and are enriched with water or biological fluids that closely resemble living soft tissues [53]. Due to its hydrophilicity, biocompatibility, and good biomechanical properties, hydrogel is always used in a wide range of applications such as tissue regeneration [54, 55], wound healing [56], and sensors [57, 58].

Many revolutionary advances have been made in the strategy of hydrogel coating of biometal substrates, and the main methods include the surface bridging method, surface initiation method, and hydrogel coating method. This article centers on the combination of hydrogel and biometal and introduces the biometal surface treatment methods, starting from the surface pretreatment, and compares the advantages and disadvantages of various methods. At the same time, the article reviews the methods and applications of hydrogel‐biometal substrate bonding in the current research.

## 2 Biometal Surface Treatment

### 2.1 Physical Modification

#### 2.1.1 Sandblasting Treatment

**FIGURE 3:**
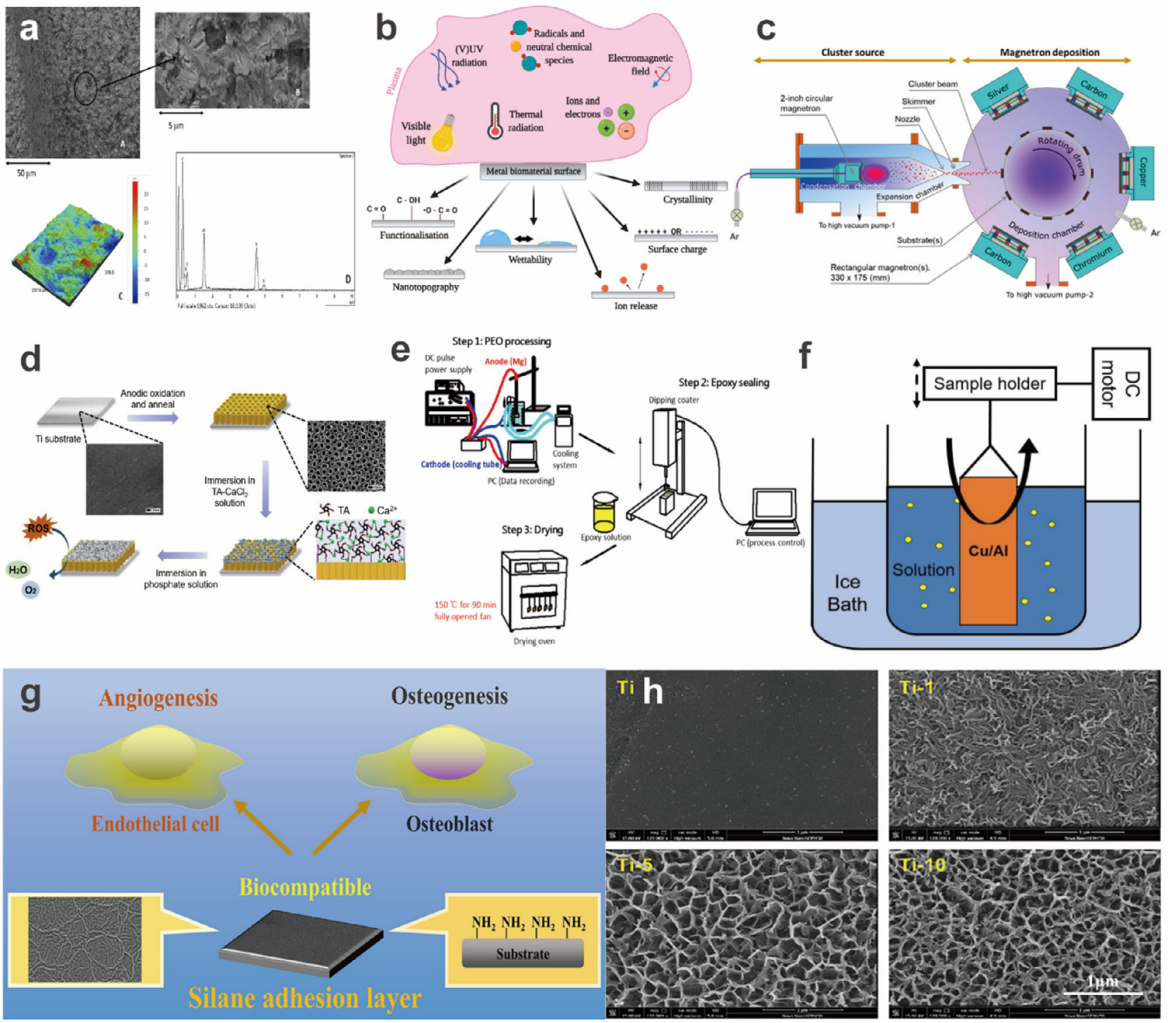
Overview of metal surface treatment applications. Physical modification: (a) Surface of a sandblasted sample using aluminum oxide as an abrasive. Reproduced with permission [28]. Copyright 2022, MDPI. (b) Schematic representation of plasma interaction with the surface of metallic biomaterials. Reproduced with permission [20]. Copyright 2021, MDPI. (c) Schematic of the vacuum system for deposition of carbon thin films by physical vapor deposition. Reproduced with permission [35]. Copyright 2022, American Chemical Society. Electrochemical modification: (d) Schematic of TiO_2_ formation by anodic oxidation. Reproduced with permission [38]. Copyright 2020, Elsevier. (e) Schematic of micro‐arc oxidation. Reproduced with permission [43]. Copyright 2018, American Chemical Society. (f) Schematic diagram of the sol–gel method. Reproduced with permission [45]. Copyright 2021, American Chemical Society. Chemical modification: (g) Schematic diagram of silanization of metal surface. Reproduced with permission [47]. Copyright 2022, Elsevier. (h) Morphology of metal surface after alkali heat treatment. Reproduced with permission [50]. Copyright 2021, American Chemical Society.

Sandblasting is one of the common methods of physical modification of metal surfaces to cope with the biological reactions faced by metals by changing the surface roughness of the metal. In addition, the residual stress on the metal surface after sandblasting can improve the mechanical properties of the metal. Sandblasting can also increase the water contact angle of the metal surface, thus increasing the surface hydrophilicity. Gil et al. [28] investigated the effect of alumina sandblasting on the physicochemical properties of titanium surfaces, and in vivo studies showed that alumina sandblasting can promote bone tissue growth. In addition, residual alumina also had a certain bactericidal effect, reducing the number of bacteria adhering to titanium (Figure 3a). Immobilization of type I collagen on sandblasted titanium surfaces using the natural cross‐linking agent kynurenic acid enabled the acquisition of surface features with hydrophilic nano/submicron pore size networks. The proposed surface modification scheme enhanced the initial cell‐surface interaction, thereby accelerating cell proliferation, initial cell differentiation, and mineralization [29]. A potential strontium‐doped titanium coating (Sr‐SLA) was prepared by sandblasting and acid etching combined with a hydrothermal method for oral implant materials. The coating has good biocompatibility, antioxidant ability, and anti‐lipogenesis, and is expected to have clinical applications in the elderly population [30]. Sandblasting can clean the metal surface, obtain different roughness, and achieve the purpose of beautification, but the disadvantages are that sandblasting is noisy, pollution is serious, and special equipment is needed. So, sandblasting is suitable for large‐scale specialized factories to pre‐treat the metal, and so on.

#### 2.1.2 Plasma Treatment

Plasma treatment can significantly improve the physical and chemical properties of biometal surfaces, such as roughness, hydrophilicity, and crystallinity. This method plays an important role in the application of biometals as medical materials in the field of biomedical engineering. Commonly used plasma treatment methods include plasma painting, plasma impregnation, ion implantation, plasma vapor deposition, plasma electrolysis oxidation, and novel surface treatment methods based on gaseous plasma [20] (Figure 3b). Bose et al. [31] used thermal oxidation for plasma spraying on Ti6Al4V substrates, which improved the crystallinity of hydroxyapatite (HA) coatings. In a rat distal femur model, the additive promoted the proliferation and differentiation of osteoblasts and significantly enhanced osseointegration and bone mineralization. On the other hand, 3d‐graphene was directly synthesized on a germanium (Ge) substrate by plasma‐assisted chemical vapor deposition using two‐dimensional graphene (2d‐graphene) as a template layer [32]. 3d‐graphene/Ge heterojunctions showed improved interactions with incident light and photogenerated charge transfer. Cisternas et al. used the plasma immersion ion implantation and deposition (PIII&D) technique to inject nitrogen ions into a titanium substrate in a capacitively coupled RF plasma. The treated titanium can be used to support stable phospholipid artificial membranes (SLBs) with good biocompatibility [33]. Compared with traditional treatment methods, plasma treatment has the advantages of high automation, high energy of reaction process, high efficiency of modification, and so on, but there are also shortcomings, such as the reaction time is not easy to control. Plasma technology is widely used in aerospace, petrochemical, automotive, and other industries for metal treatment.

#### 2.1.3 Physical Vapor Deposition

Physical vapor deposition (PVD) technology denotes the technique of depositing a thin film with a particular function on the surface of a substrate under vacuum conditions, using physical methods to vaporize the material source—a solid or liquid surface—into gaseous atoms, molecules or partially ionized into ions, and depositing them through a low‐pressure gas (or plasma) process. Gao et al. [34] applied high‐purity Mg (99.99%) to bombard and sputter the surface of the substrate to form a coating of about 1 µm magnesium grains of uniform size. The deposited Mg film had a dense and smooth surface. The magnesium‐coated Ti6Al4V stent accelerated early angiogenesis as well as osseointegration and osteogenesis around and within the stent. Thus, this new magnesium‐coated Ti6Al4V scaffold expands the range of magnesium applications and is very promising as a bone substitute for orthopedic applications. Silver‐doped, silver‐doped copper, and cluster‐doped silver amorphous carbon coatings were prepared in standard Earth gravity and microgravity environments to study the aging and antimicrobial properties of the coatings in aerospace applications. This provides a solid foundation for the further development of long‐life antimicrobial surfaces for application on manned spacecraft [35]. Surface modification was achieved by preparing coated protective electrodes using a physical deposition technique (Figure 3c) [36], where a magnetron‐sputtered aluminum‐based alloy protective layer was plated on a zinc anode. This work highlights the important role of aluminum dots in modulating the electrostatic shielding effect for stable plating and further provides insights into the design of surface protection layers for high‐performance ZIBs, as well as other potential multivalent ion batteries. The main advantages of PVD are lower processing temperatures and higher processing efficiency. The disadvantages are high equipment operation and maintenance requirements, high costs, and so on. PVD is suitable for the preparation of coatings with specific properties (electrical conductivity, wear resistance, corrosion resistance, etc.).

### 2.2 Chemical Modification

#### 2.2.1 Electrochemical Modification

##### 2.2.1.1 Anodic Oxidation

Anodic oxidation is one of the methods of electrochemical modification of metal surface, the metal or alloy to be modified as the anode, under the action of the applied current, the method of electrolysis is used to make the surface of different thicknesses of the oxide film. The metal oxide film changes the surface state and performance to achieve the purpose of surface modification, enhancement of corrosion resistance, and extension of service life. A two‐stage anodic oxidation method was able to grow nano‐anodized aluminum oxide (NAA) arrays on conductive indium tin oxide (ITO)/glass electrodes to form highly sensitive and responsive label‐free biosensors for sensitive electrochemical detection without the need for redox reactions in the electrolyte [37]. Lin et al. [38] prepared a hydroxyapatite (HA)/TA composite coating based on an anodized and annealed titanium dioxide (TiO_2_) nanotube array modified titanium substrate. The results of in vitro cytological experiments showed that the HA/TA coating had good cytocompatibility for osteoblast proliferation and adhesion (Figure 3d). On top of the material's antistatic properties, the micro‐ and nanostructured AAO not only effectively reduces the triboelectricity of the metal but also protects the sample itself, providing an excellent antistatic material option for vacuum and ungrounded environments [39].

##### 2.2.1.2 Micro‐Arc Oxidation

MAO, also known as anodic spark deposition (ASD) or plasma electrolytic oxidation (PEO), is an electrochemical treatment that forms a hard, thick, porous oxide layer on a metal substrate. Since the oxide layer grows in the direction of the substrate, the oxide layer formed by MAO has high adhesive strength [40]. Bai et al. [41] prepared microstructured and nanostructured surfaces using a micro‐arc oxidation process of pure titanium, and then annealed them at different temperatures. By varying the annealing temperature, the physical (morphology and wettability) and chemical (composites and crystallinity) properties of the coatings could be tuned simultaneously. The surfaces have favorable physicochemical properties that synergistically modulate bone immunomodulation, bone/angiogenesis, and cross‐linking between immunomodulation, osteogenesis, and angiogenesis, significantly enhancing osseointegration and thus providing an optimal strategy for surface coating of biomedical implants. TiO_2_‐hydroxyapatite (TiO_2_‐HA) coatings were able to be prepared on CP (commercially pure) and UFG (ultrafine‐grained pure) titanium by a combination of microarc oxidation and hydrothermal treatment to improve their cytocompatibility. The results showed that such coatings formed on UFG titanium were more hydrophilic and in vitro cytocompatible than conventional coatings based on CP titanium. The higher hydrophilicity and in vitro cytocompatibility of the coatings formed by heat treating MAO with water compared to using only MAO highlights the great potential of UFG TiO_2_‐HA coatings for orthopedic and dental applications [42]. In addition to this, the ability to deposit epoxy‐based polymers as sealers on porous anodic oxide coatings prepared by plasma electrolytic oxidation (PEO) to build multilayered “soft and hard” coatings on magnesium substrates. The epoxy polymer sealed micropores and other defects in the anodic oxide layer, thereby reducing the surface roughness (Figure 3e) [43]. The abrasion resistance of pure magnesium (Mg) was also improved using the micro‐arc oxidation (MAO) technique [44]. The Cu coatings exhibited potent antimicrobial activity against *Staphylococcus aureus* and enhanced osteogenesis and angiogenesis in vitro.

##### 2.2.1.3 Sol–Gel Approach

Under certain conditions, a salt solution is used as a reaction material to generate a sol–gel solution through hydrolysis and condensation, and when the solution becomes a sol–gel, the sol–gel is coated on the metal substrate to be protected, and the coating on the surface is cured and densified after drying and heat treatment, which is known as the solution‐gel method. This method enables the introduction of multifunctional coatings on metal surfaces. For thermal systems, thick coatings are undesirable because the increased thermal resistance of the coating leads to a reduction in overall heat transfer. Therefore, the development of ultrathin (<10 µm) coatings is desirable. Khodakarami et al. [45] developed a sol–gel solution that enables easy and controlled dip coating on arbitrary metals to form very smooth (<5 nm roughness), thin (∼3 µm), and conformal dense SiO_2_ coatings. This work not only demonstrates scalable coating methods for the application of ultra‐thin anticorrosion coatings but also develops a mechanistic understanding of corrosion mechanisms on a variety of functional surfaces and substrates. Sol–gel methods are often combined with other methods to achieve metal surface modification (Figure 3f). The sol–gel method allows for the doping of Ta_2_O_5_ into titanium substrates, followed by the use of micro‐arc oxidation to cure the Ta_2_O_5_. Sol–gel‐assisted micro‐arc oxidation creates a hierarchical, rough structural morphology on the titanium substrate. Strontium ions are introduced by the MAO technique, which results in a hierarchical, rough structural morphology with a large number of crater‐like micro‐pores. Based on these results, sol–gel‐assisted micro‐arc oxidation is a new strategy for introducing tantalum into implant membranes. This feature makes it a good method for fabricating bioactive coatings on implants and bone repair and regeneration scaffolds [46].

#### 2.2.2 Surface Silanization

Surface silanization is a surface treatment technology using silane coupling agents as the main raw material, capable of grafting stable chemical bonds on metal surfaces. However, a single silane layer is fragile and usually needs to be combined with other modification methods. The silane modifier (X(CH_2_) nSiR_3_) has two different functional groups. The organic functional group (X) is chosen for its reactivity or compatibility with organic materials. Hydrolyzable groups (R), such as methoxy, ethoxy, and so on, are intermediates for the formation of silanol groups for binding to inorganic or NPs surfaces. Zhao et al. [47] successfully prepared silane adhesion layers of different thicknesses, integrity, and surface morphology by introducing 3‐aminopropyltriethoxysilane on the surface of alkali‐treated titanium during the silanization process. The results of the in vitro evaluation showed that extending the silanization treatment time increased the thickness and integrity of the silane adhesion layer and significantly improved its biocompatibility. This work systematically investigated the biocompatibility and biological functions of the modified silane adhesion layer, providing a valuable reference for its application on biomolecular covalent transplantation (Figure 3g). Ladeira et al. [48] investigated the effect of processing Al_2_O_3_ NPs with vinyltrimethoxysilane (VTMS) on the properties of high‐density polyethylene (HDPE). The authors reported that functionalized Al_2_O_3_ NPs with VTMS improved the thermal stability of HDPE nanocomposites and improved adhesion to the matrix, while masterbatch processing techniques promoted better dispersion of alumina in the HDPE matrix. In terms of metal electrode protection, Wang et al. [49] devised a simple but feasible method to achieve SEI layer enhancement and enhanced air stability by modifying lithium metal with an inexpensive silane coupling agent, 3‐methacryloxypropyltrimethoxysilane (MPS). The modified layer protects the lithium metal from air corrosion, which will reduce the high requirements for atmospheric protection in lithium metal‐based battery manufacturing. The layer can also act as a promoter to enhance the adhesion between the SEI layer and the Li substrate by forming chemical bonds and physical intertwining effects. As a result, the air stability of the Li electrode is improved, and the electrochemical performance is enhanced.

#### 2.2.3 Alkali Heat Treatment

The alkali heat treatment method is a modification technique in which metals such as titanium and tantalum are placed in a strong alkali solution for some time and then removed and post‐treated at high temperatures to form a biologically active surface. By introducing 3‐aminopropyltriethoxysilane on the alkali‐treated titanium surface during the silanization process, silane adhesion layers with different thicknesses, integrity, and surface morphologies were successfully prepared. In vitro evaluation showed that prolonging the silanization treatment time increased the thickness and integrity of the silane adhesion layer and significantly improved its biocompatibility (Figure 3h) [50]. Treatment of Al_2_O_3_ NPs with VTMS was able to enhance the thermal stability of the HDPE nanocomposites and improve the adhesion to the matrix, while the masterbatch processing technique promoted better dispersion of alumina in the HDPE matrix. For metal electrode protection, enhancement of the SEI layer and improved air stability could be achieved by modifying lithium metal with an inexpensive silane coupling agent, 3‐methacryloxypropyltrimethoxysilane (MPS) [51]. Based on alkaline heat treatment, Tan et al. [52] developed a food‐grade probiotic‐modified implant to prevent methicillin‐resistant *S. aureus* infection and accelerate osseointegration.

## 3 Methods of Coating Biometal Surfaces With Different Hydrogels

**FIGURE 4:**
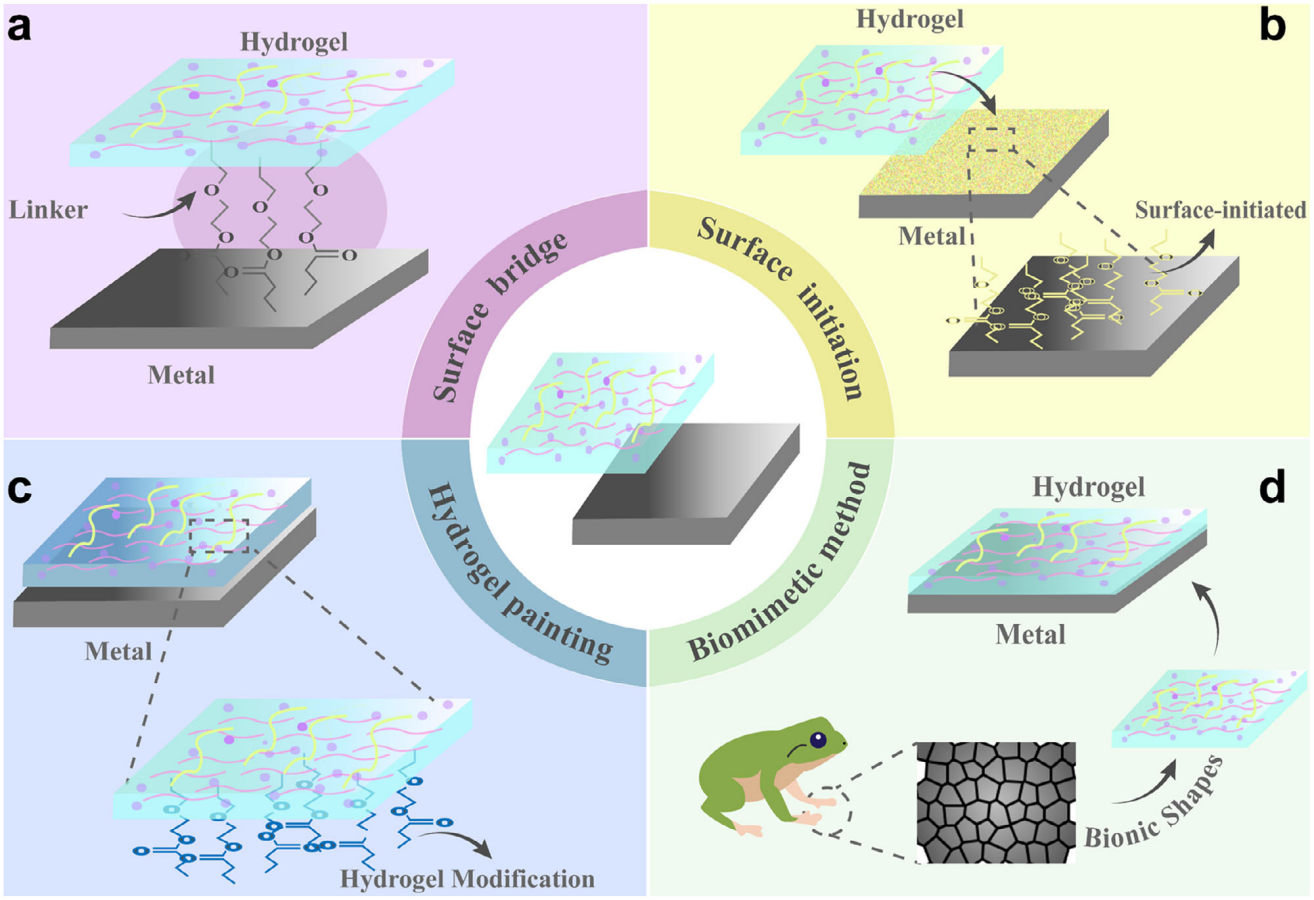
Biometal‐hydrogel bonding methods. Principles and diagrams of (a) the surface bridging method, (b) the surface initiation method, (c) the hydrogel coating method, and (d) the biomimetic method.

After the modification of the metal surface, the next step is the preparation of hydrogel coatings on the biometal surface. The existing hydrogel‐biometal bonding methods include the surface bridging method (Figure 4a), the surface initiation method (Figure 4b), the hydrogel coating method (Figure 4c), the biomimetic method (Figure 4d), etc. No matter which method is used, the aim is to form a coating with high bonding strength to the biometal surface and adaptability to different surfaces (Table 2). The different methods will be described below.

**TABLE 2:** Comparison and application of methods for bonding hydrogels to biometal surfaces.

Method	Biometal	Hydrogel	Advantages and disadvantages	Application	References
The surface bridge method		Polyvinyl alcohol, chitosan, polyacrylamide, etc.	Realization of strong bonding of hydrogels to metal surfaces. Few types of bridging molecules, a complex modification process.	Suitable for biometals and hydrogels with easy‐to‐treat surfaces.	[59, 60, 61, 62]
The surface initiation method	Titanium, Ti6Al4V, etc.	HA‐based hydrogel coatings, CHI‐based hydrogel coatings, polyethylene glycol, etc.	Wide range of applications and simple processing steps. Waste of large amounts of hydrogel precursors.	Suitable for forming hydrogel coatings on most biometals.	[63, 64, 65]
The hydrogel paint method	ZnO, Ti, steel, Al, etc.	GelMA, HAMA, polycaprolactone, polyacrylamide, etc.	Not limited by biometallic shape. Only for single network hydrogels, poor mechanical properties.	Suitable for coating hydrogels on large biometal surfaces.	[66, 67, 68]
The biomimetic method	Stainless steels, Ni, etc.	N‐Isopropylacrylamide, N‐Phenethyl methacrylamide, poly(ethylene glycol) dimethacrylate, etc.	Hydrogels can form strong adhesion with biometals through biomimetic microstructure. Complex operating procedures and high technical requirements.	Ideal for situations where small‐scale customization is required.	[69, 70, 71]

### 3.1 The Surface Bridge Method

**FIGURE 5:**
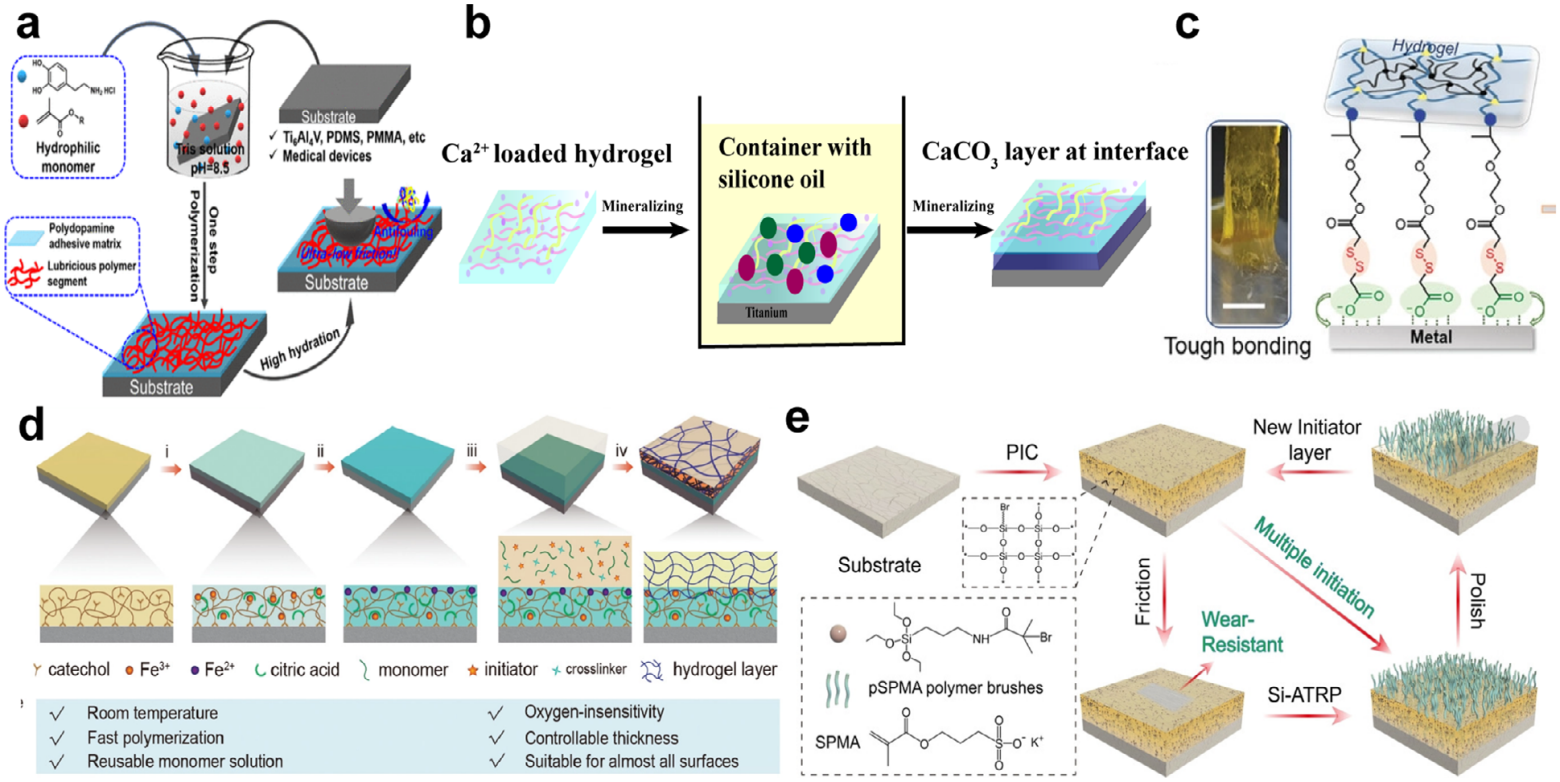
Example diagram of biometal‐hydrogel bonding methods (I). (a–c) Different bridging molecules mediate the binding of hydrogels to biometal surfaces. Reproduced with permission [81]. Copyright 2019, American Chemical Society. Reproduced with permission [84]. Copyright 2019, John Wiley and Sons. The bridging molecules used are (a) dopamine, (b) calcium carbonate, and (c) carboxylic acid group and methacrylic acid group, respectively. (d,e) Examples of surface initiation methods. (d) UV‐triggered surface‐catalyzed initiated radical polymerization (UV‐SCIRP) initiated by a sticky initiator layer (SIL) (SIL@UV‐SCIRP). Reproduced with permission [85]. Copyright 2022, John Wiley and Sons. (e) Construction of inorganic hybrid persistent (ATRP) initiator coatings on solid surfaces. Reproduced with permission [86]. Copyright 2022, John Wiley and Sons.

Due to the nature of the hydrogel itself and the presence of a large amount of water in the surrounding environment, it is difficult for the hydrogel to bond with the biometal surface by simple adhesion. At this time, if there is a bridging molecule, it can realize the strong bonding of hydrogel and biometal surfaces. The principle of strong adhesion by surface bridging is that the two ends of the bridging molecule form a strong interaction with the hydrogel and the substrate, respectively, connecting downward with the biometal substrate and upward with the hydrogel to achieve strong adhesion. Silane coupling agents are commonly used as bridging molecules in current research [72, 73, 74]. For example, Yuk et al. [59] used a simple silane modification method to achieve tough, transparent, and conductive bonding of hydrogels with biometals such as aluminum and titanium, with interfacial toughness values exceeding 1000 J m^−2^, which is better than the toughness of tendon‐bone and cartilage‐bone interfaces. This ability to fabricate extremely strong hydrogel‐solid hybrid materials makes many future research directions and applications possible. In addition, Wu et al. [75] formed an n‐haloamine polymer coating on the titanium surface with long‐lasting, renewable antimicrobial effect, stability, and biocompatibility by silane modification. Another more commonly used binder is polydopamine [76, 77, 78, 79, 80], where dopamine molecules self‐polymerize to form a PDA matrix that confers generalized adhesion to the coating on the material surface, while dopamine‐triggered in situ polymerization of monomers produces low‐friction polymer chain segments that avoid the complex synthesis of functional polymers before encoding. Wei et al. [81] used a one‐step co‐deposition strategy using dopamine and hydrophilic monomers to fabricate ultra‐low friction coatings on the surface of biomaterials (Figure 5a). The coatings exhibited ultra‐low friction properties as well as hydrophilic and anti‐fouling properties. Typical PDA‐PSBMA coatings exhibit extraordinary lubricity in pure water and biofluids down to 0.003 µm. With simple fabrication, substrate independence, and excellent capabilities, this versatile coating is considered to have a wide range of applications in biomedical devices and implants, including contact lenses, catheters, endoscopes, and so on. Researchers have also used different “binders” as bridging molecules between the hydrogels and biometal substrates, such as Jiang et al. [60] who used a double hydrogen bonding network to enable the hydrogels to adhere to the surfaces of biometals such as 316L stainless steel and aluminum, and Choi et al. [82] also used hydrogen bonding to synthesize the hydrogels. The imidazole group of the synthetic hydrogel is highly adherent to substrates such as glass and bone through multiple hydrogen bonds and does not require any chemical modification of the substrate. This approach provides an unprecedented hydrogel to mimic natural tendons and their interface with the bone, which may be useful for biomedical applications. Zhao et al. [83] used an in situ mineralization method by CO_2_ diffusion to fabricate a CaCO_3_ layer as a bridging layer at the hydrogel‐solid titanium substrate interface (Figure 5b). Zhu et al. [61] combined poly(allylamine hydrochloride) (PAH) hydrogels and ethyl α‐cyanoacrylate (ECA) as adhesives and found that they could firmly bond PVA‐glycerol hydrogels to stainless steel substrates, and the combined use of PVA‐glycerol hydrogels and ECA adhesives has promising applications in marine antifouling. Xu et al. [62] successfully introduced iron‐chitosan complexes into the hydrogel system to obtain tough, adherent, and conductive chitosan‐polyacrylamide double network (CS‐PAAm DN) hydrogels. CS‐PAAm DN hydrogels exhibited excellent adhesive behavior on different material surfaces such as biometals, glass, pigskin, rubber, plastics, and various biological tissues. In addition, researchers have innovatively designed bridging molecules, such as Li et al. [84] who designed bridging molecules containing carboxylic acid groups bound to biometal surfaces and methacrylic acid groups chemically linked to polymeric hydrogel networks and achieved modification of hydrogels with disulfide groups to bond with common biometal substrates including nickel (Ni), aluminum (Al), tantalum (Ta), silver (Ag) stainless steel, titanium (Ti), and some alloys (Figure 5c).

### 3.2 The Surface Initiation Method

Surface initiation is in principle suitable for the formation of hydrogel coatings on most substrates. For biometallic substrates, an initiator containing an initiator, such as polyvinyl acetate, can be applied to the target surface to introduce the initiator. Hydrogel coatings were prepared on the surface by introducing initiators to the biometal surface for surface‐initiated polymerization.

Among the surface initiation methods, researchers usually use the surface catalytically initiated radical polymerization (SCIRP) method, the atom transfer radical polymerization (ATRP) method, etc. The SCIRP method requires the doping of biometal catalysts into the substrate to initiate free radical polymerization [85, 87, 88]. Among them, Xu et al. [85]. proposed an innovative method of UV‐triggered surface catalyzed‐initiated radical polymerization (UV‐SCIRP) from a tacky starting layer (SIL) (SIL@UV‐SCIRP), by which the surface of almost any substrate (natural or artificial material) can be modified by a hydrogel coating with controlled thickness and diverse composition (Figure 5d). The hydrogel coating has good interfacial adhesion to the substrate and can easily modify its wettability and lubricity properties. The ATRP method is a method for constructing inorganic hybrid initiator coatings on solid surfaces, and recent advances in ATRP have addressed many of the challenges in the preparation of polymer‐modified surfaces [86, 89, 90, 91, 92, 93]. Notably, Liu et al. [86] developed an innovative and integrated method for constructing inorganic hybrid persistent (ATRP) initiator coatings (PIC) and polymer brushes (PB) on solid surfaces (Figure 5e). PIC plus PB can not only withstand 10,000 friction cycles under high pressure but also re‐initiate new ATRP reactions when the grafted PB on the PIC surface wears off. PIC can be coated on a variety of substrates, including glass, polymers, biometals, and so on. Large‐scale PIC technology can provide a viable pathway for the practical application of surface‐grafted PB. Olmo et al. successfully prepared hyaluronic acid‐based hydrogel coatings [63] and chitosan‐based hydrogel coatings [64] on Ti6Al4V surfaces by modifying the surface with phosphonate self‐assembled monolayer and thus triggering the grafting of hydrogels, which have excellent antimicrobial properties and can effectively combat the infection problems caused by implants. In addition, researchers have innovated surface initiation strategies through mimicry [65, 94]. Su et al. [65] successfully synthesized a novel mimicry surface adhesion initiator by coupling 3,4‐dihydroxyphenylacetic acid with thermal 2,2′‐azobis (2‐methyl propionamide) dihydrochloride. The synthetic initiator (DOPV) can adhere to the surface of various materials in a mussel‐inspired manner and initiate surface graft polymerization. The hydrogel coating exhibited in vitro protein resistance, anti‐biofilm efficacy, hemocompatibility, and low cytotoxicity. This simple, inexpensive, and versatile mussel‐inspired surface grafting approach provides a promising strategy for the preparation of effective antimicrobial and anti‐biofilms for the prevention of biomedical device‐/implant‐associated infections.

### 3.3 The Hydrogel Paint Method

**FIGURE 6:**
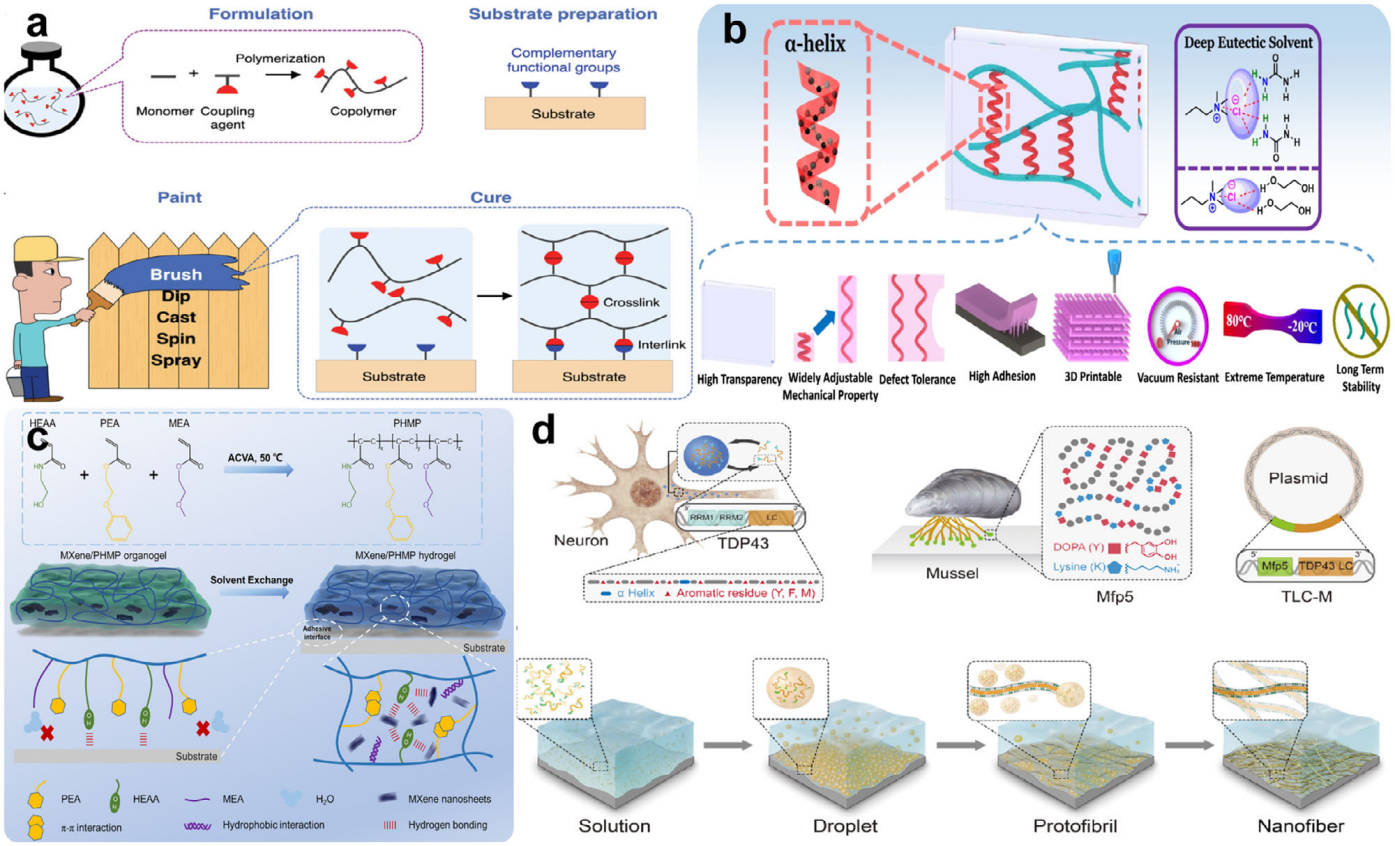
Example diagram of biometal‐hydrogel bonding methods(II). (a,b) Examples of the hydrogel coating method: (a) Principles and steps of hydrogel painting. Reproduced with permission [95]. Copyright 2022, John Wiley and Sons. (b) Schematic structure and properties of peptide‐enhanced eutectic gel coatings formed using peptides as cross‐linking agents. Reproduced with permission [96]. Copyright 2022, Springer Nature. (c,d) Examples of biomimetic method. (c) MXene/PHMP hydrogel coating inspired by barnacle proteins. Reproduced with permission [97]. Copyright 2019, American Association for the Advancement of Science. (d) Preparation of hydrogel coatings containing Mfp5 protein inspired by adhesion plaques of marine mussels. Reproduced with permission [98]. Copyright 2022, American Chemical Society.

The hydrogel coating method involves applying various hydrogels to various substrates and realizing bonding with the substrate through cross‐linking of the hydrogels. The coating method is not limited by the complexity of the substrate, thus enabling the application of hydrogel coatings to biometal substrates of various materials and shapes. The principle is divided into four parts: formulation, substrate preparation, coating, and curing. During the formulation process, monomers and coupling agents are dissolved in water, along with various other functional compounds such as initiators, chain transfer agents (CTAs), rheology modifiers, charge carriers, drugs, and toughening agents. Monomers and coupling agents form non‐cross‐linked copolymer chains through free radical polymerization reactions. During the substrate preparation, functional groups complementary to the coupling agent are given on the substrate surface. During the spraying process, the coating—an aqueous solution of the uncrosslinked polymer chains and various other compounds—is applied to the prepared substrate. During curing, the coupling agents react with each other to cross‐link the polymer chains into a polymer network and react with complementary functional groups to interconnect the polymer network with the substrate. The hydrogel coating method has greatly reduced the difficulty of preparing coatings on biometal surfaces and has inspired many coating manufacturers and coating users to invent hydrogel coatings for industrial and everyday functions [95] (Figure 6a).

In the hydrogel coating method, researchers usually use adhesive molecules modified hydrogel to achieve the adhesion of hydrogel to the substrate, including mussel‐inspired catechol groups [66, 67, 68, 99, 100, 101, 102, 103, 104], tannins [105, 106, 107, 108], silane coupling agents [109], casein [110], peptide cross‐linking agents [96] (Figure 6b), etc. In addition, researchers also use electronically controlled click [111] and annealing crystallization [112] to achieve biometal surface‐bound hydrogel.

### 3.4 The Biomimetic Method

In the past few years, exciting advances have been made in the field of bionic adhesion, which is inspired by natural adhesion mechanisms. These biomimetic hydrogels can form strong adhesions to substrates through chemical reactions or surface microstructures. Many organisms, such as sandcastle worms [69], mussels [70], and barnacles [98, 113, 114] (Figure 6c), achieve binding of hydrogels to biometal substrates through catechol chemistry, complex cohesions, and cation‐π interactions [115].

Microstructural adhesive systems in nature are equally impressive, with researchers finding inspiration from structures such as sticky fish adhesive discs [116], octopus suction cups [117], and tree frog feet [71], for example, Zhang et al. were inspired by the surface structure of tree frog foot pads to design a bionic hydrogel that adheres and detaches on‐demand on a variety of material surfaces, which is achieved through switchable, thermally triggered hexagonal micropillar‐patterned hydrogels on shape transformation to achieve this. Due to dynamic hydrogen bonding and dipole–dipole interactions in the hydrogel, the hydrogel exhibits thermally triggered shape memory effects. The hydrogel can recover more than 15% of its shape within 80 s. Based on the shape memory effect of hydrogels, the adhesion strength can be changed by thermal stimulation. The adhesion strength of the microstructure recovered from the hydrogel surface was reduced to 15.4% of the initial adhesion strength. The switchable underwater adhesion of hydrogels can be used in transfer printing, medical adhesives, mobile robotics, and so on. In addition, some researchers design hydrogels for bonding to biometal substrates by simulating structures such as microstructure or nanostructure arrays of biogenic fiber adhesives [118] and mammalian low‐complexity structural domains [97] (Figure 6d). This biomimetic type of bonding is based on physical interactions that create an immediate bond and no chemical residues on the adhesive substrate. This approach has great potential for sustainable medical materials.

## 4 Applications and Functions Brought by Hydrogel Coating on Biometal Surfaces

### 4.1 Sensing Film

**FIGURE 7:**
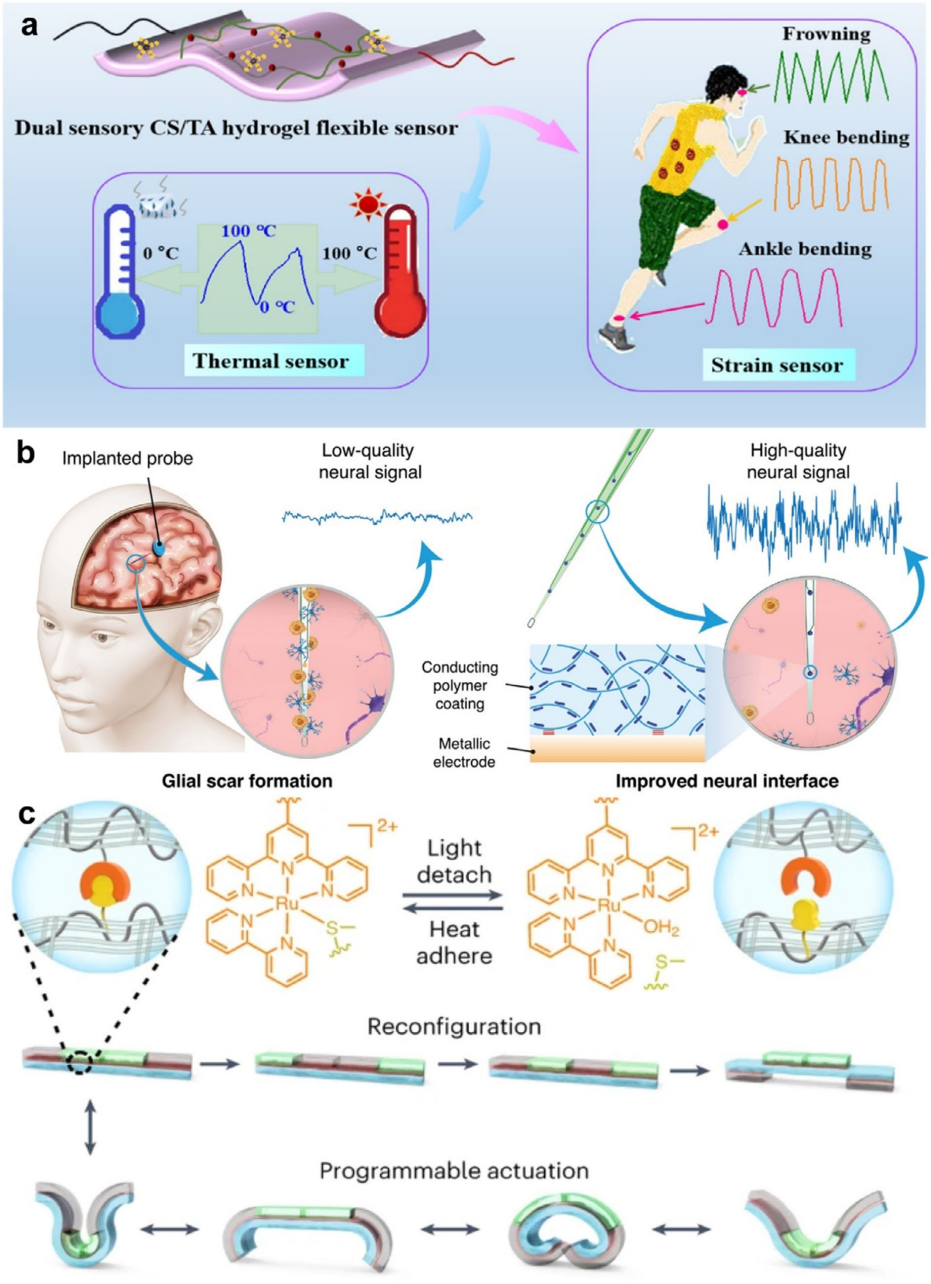
Hydrogel coating applications on biometal surfaces (non‐disease component). (a) Hydrogel coatings are used to accurately monitor human motor behavior, differentiate physiological signals, and recognize speech. Reproduced with permission [123]. Copyright 2022, American Chemical Society. (b) Engineering electrodes with robust conducting hydrogel coating for neural recording and modulation. Reproduced with permission [129]. Copyright 2022, John Wiley and Sons. (c) Hydrogel adhesives that can accommodate drive and shape changes in implanted robots. Reproduced with permission [130]. Copyright 2024, Nature.

Hydrogels are candidates for manufacturing flexible sensors because of their softness, flexibility, and satisfactory stretchability. Flexible hydrogel sensors exhibit stable electrical conductivity and strain sensitivity to monitor both macroscopic and fine human motion [119]. Biodegradable sensors represented by hydrogels can be used as implantable sensing devices with good biosafety and are also the most feasible and desirable solution to the problem of hard‐to‐treat electronic waste (i.e., e‐waste) [120]. Recent work of researchers has proposed different strategies to prepare hydrogels on biometal surfaces as sensing membranes, which are promising in the preparation of flexible sensors for wearable applications. Different materials of hydrogel sensing membranes impart different properties to the sensors [98, 107, 108, 121, 122, 123, 124] (Figure 7a), such as Makhsin et al. [125] proposed a method to improve the sensitivity of the metal‐clad leaky waveguide sensors by incorporating amine‐reactive functional groups in low refractive index acrylate‐based polymers. Cui et al. [126] proposed high‐strength, stretchable, compliant, and self‐adhesive chitosan/poly(acrylic acid) dual network composite hydrogels for epidermal strain sensors. Uzair et al. [127] designed an epoxy polyethylene glycol pH sensor film that can be directly implanted or coated on the surface of orthopedic implants to provide high spatial resolution tissue pH maps by X‐ray excited luminescence chemical imaging (XELCI). This is particularly useful for detecting local pH changes during the treatment of implant‐associated infections. Mugo et al. [128] constructed a robust microneedle microneedle microextraction platform for chromatographic and dual electrochemical detection by a layer‐by‐layer assembly method. The platform was able to detect the precise amount of caffeine in food and beverages with stability. This idea broadens the application scope of hydrogel sensors and provides new ideas for further development of hydrogel sensor membranes on biometal surfaces.

### 4.2 Electrode Coating

Conventional biometal bioelectrodes have many limitations, such as the inherent mismatch between rigid electrodes (i.e., silicon and tungsten, modulus over 100 GPa) and soft brain tissue (modulus below 1 kPa), and a long‐term robust conformal interface between conventional electrodes and brain tissue remains difficult to achieve. As a result, long‐term electrode implantation is always accompanied by scar formation and inflammatory reactions, which significantly impair the implantation effect. Recent developments in soft electronics and micro/nanofabrication open new paths to overcome these challenges. Hydrogels can be used to condition metal anodes in electrolyte engineering due to their multifunctional properties, such as interaction with water‐solubilized structures, formation of in situ interphase layers, and so on, which are effective in extending the service life of metal electrodes [131, 132].

Zhang et al. [129] established a versatile and reliable method to design robust conductive hydrogel coatings on conventional biometal bioelectrodes (Figure 7b). The simplified fabrication method involves chemical grafting of functionalized long‐chain polymers (polystyrene sulfonic acid‐*co*‐4‐vinylpyridine), poly (SS‐4VP)) onto a biometal substrate, followed by electrochemical deposition of conducting polymers (i.e., PEDOT) and chemical cross‐linking to produce a PEDOT: poly (SS‐4VP) interpenetrating network. In addition to the ability to form conformal interfaces with rigid electrodes, the coating has highly desirable electrical conductivity and long‐term electrochemical stability, enabling high‐quality recording of electrophysiological signals. Silva et al. [111] by indirectly controlling click chemistry reactions, we assembled well‐defined covalent cross‐linked gels on gold, platinum‐iridium (PtIr), indium tin oxide (ITO) planes, and separate structures such as electrode tips. This approach addresses a key bottleneck in integrating bioactive materials with electronic conductors. Zhu et al. [133] reported a polyimide coating to solve the corrosion problem. The coating prevents corrosion, thereby reducing the capacity loss of the standby microcell to 10%. Coordination of carbonyl oxygen with zinc ions in the polyimide builds up during cycling, forming a zinc layer that minimizes the concentration gradient through the electrode/electrolyte interface, resulting in fast kinetics and low plating/stripping overpotential.

### 4.3 Robot Implantation

Currently, there is significant interest in the development of implantable robotic devices to enable patients suffering from severe hormonal alterations or chronic pain to benefit from the automatic and effective administration of specific medications. However, there are specific challenges associated with the long‐term use of electronic implants, including stable performance and poor material bio‐ and cytocompatibility, leading to immune reactions and infections. For implantable materials, it is important to address the interface between the device and the body. For better interaction between the medical device and its surrounding biological systems, polymer coatings are often applied [134]. Hydrogel coatings can be adapted to the implanted robot's actuation and shape changes, and can also be adjusted to achieve programmability and customizability by adjusting the hydrogel composition (Figure 7c) [130]. Jing et al. [135] developed a class of soft robot skins based on two‐dimensional materials (2DM) and gelatin hydrogels with skin‐like versatility (stretchability, temperature regulation, threat protection, and strain sensing). 2DM‐integrated hydrogel (2DM/H) skins enable soft robots to perform specified tasks at high temperatures and under various environmental threats while maintaining a mild machine temperature. Bernasconi et al. [136] proposed a method to achieve slow drug release for targeted micro‐robotics using a layer‐by‐layer coating process to coat alginate hydrogels on magnetically guided microdevices by positive and negative charge binding. At the same time, the biocompatibility and lifetime of the devices were improved. This method has great potential for in vivo targeted drug delivery robotic applications. Özkale et al. [137] proposed a method to fabricate bio‐inspired soft microelectromechanical system devices. The strategy combines techniques such as programmable colloidal self‐assembly, plasmonic nanosensor light harvesting, and in situ polymerization consistent with the hydrogel mechanism. Opto‐mechanical microactuators were synthesized using a template‐assisted microfluidic approach in which gold nanorods were coated with thermosensitive poly(*N*‐isopropylmethacrylamide) (pNIPMAM) polymers as nanoscale building blocks. The prepared microactuators have mechanical properties and performance indexes comparable to bioengineered muscle structures.

### 4.4 Disease Treatment

#### 4.4.1 Bone Implant Coatings

**FIGURE 8:**
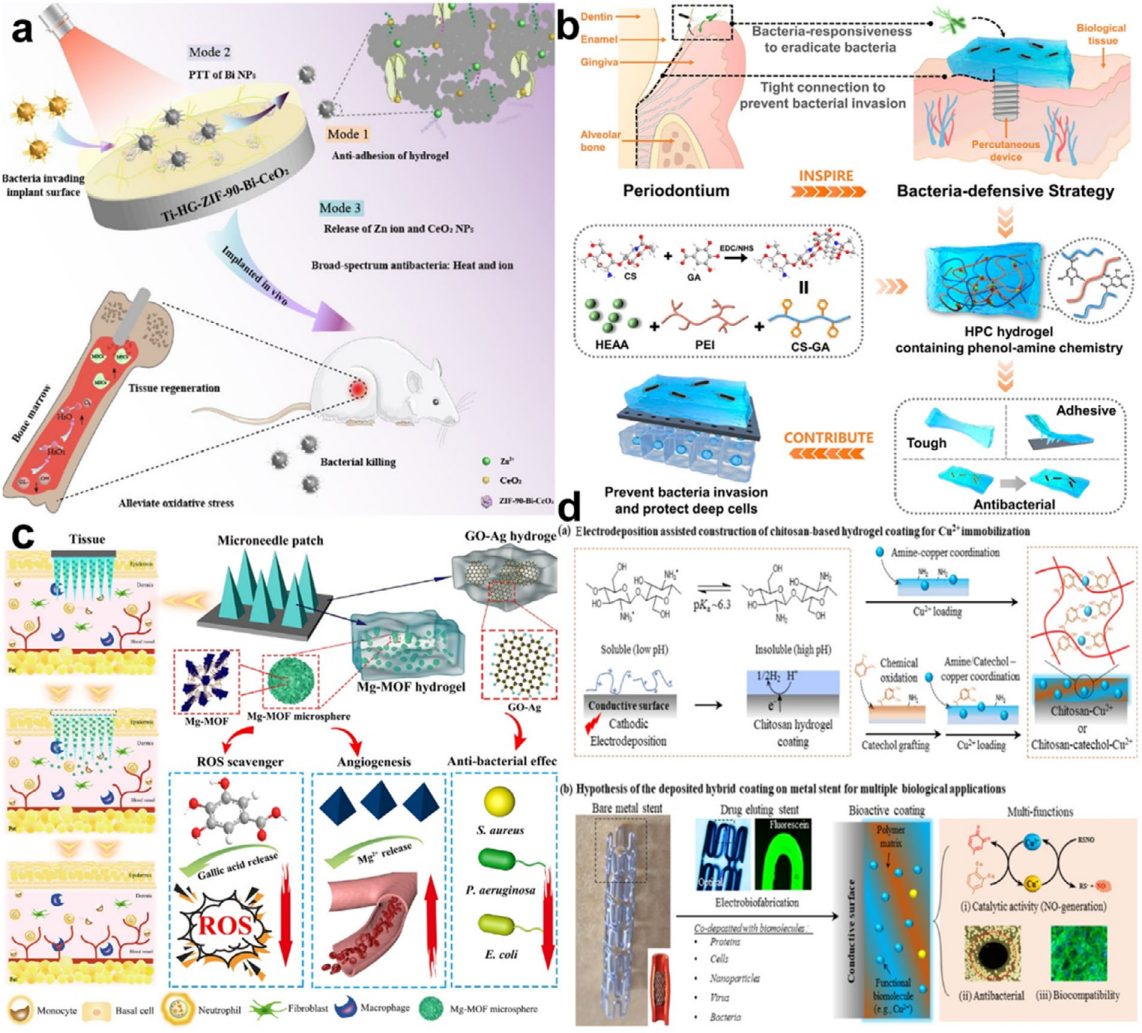
Hydrogel coating applications on biometal surfaces (disease‐oriented). (a) Hyaluronic acid/gelatin nanocomposite hydrogel coating on titanium‐based implants for the treatment of biofilm infections and hyperinflammatory responses. Reproduced with permission [141]. Copyright 2023, American Chemical Society. (b) Antimicrobial hydrogel coating for periodontitis treatment. Reproduced with permission [146]. Copyright 2022, American Chemical Society. (c) Multifunctional magnesium organic skeleton microneedle patch for accelerated diabetic wound healing. Reproduced with permission [147]. Copyright 2021, American Chemical Society. (d) Catechol‐grafted chitosan coatings as an efficient and multifunctional platform for immobilization of bioactive molecules (e.g., copper ions) and surface modification of biometallic medical devices. Reproduced with permission [101]. Copyright 2021, Elsevier.

Implant‐associated infections (IAI), caused by pathogens colonizing the implant surface, are a serious problem in traumatic orthopedic surgery and often lead to implant failure. Complications of IAI are usually accompanied by significant financial costs and long hospital stays, posing a great threat to the clinical practice of implants [138]. Hydrogels resemble the porous structure of natural tissues and promote cell adhesion, proliferation, and differentiation. In the field of bone implants, it is possible to combine osteogenic properties with antimicrobial, immunomodulatory, and pro‐angiogenic functions by designing hydrogel coatings with specific functionalities [139, 140]. Researchers have developed different surface coatings for bone implants to achieve antimicrobial [138, 141] (Figure 8a), osteogenic [142, 143], and anti‐corrosive effects [144] while achieving controlled drug release [145], which has solved the current problems of bone implants. For example, Ding et al. [141] fabricated a novel photothermally enhanced bifunctional hydrogel coating system on Ti implants. The modified Ti substrate exhibited multi‐modal antibacterial and anti‐biofilm functions, which were attributed to the photothermal effect of Bi NPs and the release of Zn^2+^ ions and CeO_2_ NPs. This strategy overcomes the shortcomings of traditional approaches, focusing only on anti‐infective treatment, and points to a research direction focusing on host microenvironment regulation. It will provide new ideas for the treatment of biofilm infections and excessive inflammation.

#### 4.4.2 Dental Material Coatings

Microbial accumulation on dental materials poses a significant risk for oral disease and leads to the deterioration of material properties. Therefore, antimicrobial materials that resist microbial colonization and eliminate pathogenic bacteria have shown outstanding advantages in restoring dental health [148]. Researchers have designed hydrogel coatings of different compositions for dental orthodontics [149], transparent dental laminates [91], dental implants [146, 150] (Figure 8b), and other dental materials to achieve antimicrobial, anti‐carious, and guided periodontal tissue regeneration. Inspired by periodontal disease, Sun et al. developed a bacterial defense hydrogel containing phenolamine chemistry. To introduce phenol‐amine chemistry into the hydrogel and impart antibacterial properties, CS with inherent antibacterial properties was modified with gallic acid (GA). HEAA/PEI/CS‐GA (HPC) hydrogel exhibited excellent toughness, universal adhesion, and superior antibacterial properties. In addition, the reported HPC hydrogels perfectly demonstrate bacterial defense and are considered emerging, versatile materials for the prevention of bacterial infections and the protection of deep tissues.

#### 4.4.3 Diabetes Mellitus

Currently, research on hydrogel coatings on biometal surfaces is very popular in the treatment of diabetes‐related diseases. Persistent hyperglycemia and chronic metabolic dysfunction in diabetic patients lead to a cycle of inflammation that continues to compromise the patient's innate and adaptive immune systems, resulting in a variety of complications. By functionalizing the hydrogel coating, it is possible to provide the coating with precise antimicrobial, diabetic microenvironment regulation, and immune modulation functions [151]. In the work of Tong et al. [152] insulin was loaded onto inorganic nanosheets (layered double hydroxides, LDHs) and coated on copper wires by electrodeposition with chitosan. The structures formed in the chitosan composite hydrogels effectively encapsulated insulin and were validated by various techniques. In vivo, experiments in diabetic rats showed that controlled insulin release in plasma and steady reduction of blood glucose could be achieved by using appropriate electrical signals. Yin et al. [147] developed a microneedle patch based on a magnesium organic framework (denoted MN‐MOF‐GO‐Ag) that enables transdermal drug delivery and combination therapy for diabetic wound healing. The released Mg^2+^ induces cell migration and endothelial tubule production, while gallic acid is a reactive oxygen scavenger that promotes antioxidation (Figure 8c). Mice treated with MN‐MOF‐GO‐Ag achieved a significant improvement in wound healing. Zhao et al. [153] used oxidized dextran (OD) and phenylboronic acid‐functionalized polyethyleneimine (PBA‐PEI) to develop a novel injectable topical drug delivery system (LDDS) to achieve ROS‐triggered local drug release by B‐N landing with simultaneously improved drug delivery efficiency (doxycycline [Doxy] and metformin [Met]). Dual‐loaded PBA‐PEI/OD hydrogel may be a favorable potential candidate for CPDM management in the dental clinic as a novel therapeutic agent.

#### 4.4.4 Cardiovascular Disease

Hydrogels also have many applications in the field of cardiovascular disease due to their excellent blood and cell biocompatibility, adjustable mechanical properties, and other characteristics. By functionalizing hydrogel coatings, it is possible to provide cardiovascular devices with anticoagulation and rapid endothelialization [154]. For coronary stent coatings, Wang et al. [101] reported a green and simple electro‐fabrication method to construct bioactive hydrogel coatings by combining chitosan, catechol moieties, and copper ions on coronary stents and titanium surfaces (Figure 8d). Wen et al. [72] developed a biocontamination‐resistant and endothelium‐selective amphoteric hydrogel coating to simultaneously enhance the titanium surface's nonspecific resistance and rapid endothelialization. For vascular cannulation hemostasis, Wei et al. [155] coated a hydrogel composed of sodium alginate, hyaluronic acid, and calcium carbonate onto the surface of a suture needle. The hydrogel‐coated hollow needle can effectively and rapidly stop bleeding from puncture sites in solid organs and blood vessels. Similarly, Ren et al. [156] prepared hemostatic needles based on hydrogels by coating the surface of syringe needles with alginate‐CaCl_2_ with carefully optimized mechanical and chemical properties to effectively prevent bleeding after tissue puncture. In the treatment of cardiovascular implantable electronic device (CIED) infections, Dong et al. [157] designed an antibacterial CHA/CHX hydrogel based on HA scaffold with a hybrid cross‐linking strategy to prevent CIED pocket infections. This HA‐based antimicrobial hydrogel has excellent injectability, deformability, and persistent non‐antibiotic treatment properties and has great potential in preventing CIED pocket infections.

## 5 Conclusions and Outlooks

This paper reviews the research progress of hydrogel coatings on biometal surfaces, focusing on biometal surface pretreatment methods, hydrogel‐biometal bonding methods, and applications of hydrogel coatings on biometal surfaces. Before the hydrogel is combined with biometal, the biometal is usually pretreated. The main methods used in current research are physical, electrochemical, and chemical methods. In practice, these methods are usually used in combination to achieve the desired results of the researchers. The main hydrogel‐biometal bonding methods are surface bridging, surface initiation, hydrogel coating, and bionic methods. Hydrogel coatings on biometal surfaces have a wide range of applications in both medical and non‐medical fields.

However, the longevity, reproducibility, and multiple response properties of hydrogel coatings on biometal surfaces are still less than outstanding in current research. Researchers tend to emphasize the short‐term and immediate effects and ignore the problems of long‐term use of the coatings. Therefore, the preparation of hydrogel coatings with long‐lasting and multi‐responsive properties for biometal surfaces is a hot topic for the next research. For example, researchers have recently developed hydrogel coatings with smart response [76, 158], anti‐fatigue [159], renewable [160], and amphiphilic hydrogel properties [161]. In addition, in the hydrogel‐biometal bonding method, the current research mostly adopts the chemical method as the main means to realize the bonding with the assistance of other methods. This method often has the potential threat of toxicity. Therefore, the development of more stable and safer means of binding is the trend of future research, and some researchers have achieved good results by using bionic means to combine biometal and hydrogel [94].

As the iterations of biometals and hydrogels continue to be updated and the methods of combining them continue to advance, the application areas of hydrogel coatings on biometal surfaces will continue to expand. This expansion will occur not only in the medical field (e.g., biosensors, implantable robots, oncology therapeutic areas), but also in non‐medical areas where they can create value (e.g., ocean engineering, aerospace, etc.). At the same time, the development of safer‐acting drugs, more effective hydrogel components, and more simple and excellent biometal treatment methods has become the goal of future research, but also to promote the biometal surface hydrogel coating research toward a deeper and broader direction.

## Conflicts of Interest

The authors declare no conflicts of interest.
